# Mid-infrared hyperchaos of interband cascade lasers

**DOI:** 10.1038/s41377-021-00697-1

**Published:** 2022-01-02

**Authors:** Yu Deng, Zhuo-Fei Fan, Bin-Bin Zhao, Xing-Guang Wang, Shiyuan Zhao, Jiagui Wu, Frédéric Grillot, Cheng Wang

**Affiliations:** 1 School of Information Science and Technology, ShanghaiTech University, Shanghai, 201210 China; 2 LTCI, Institut Polytechnique de Paris, Télécom Paris, 19 place Marguerite Perey, 91120 Palaiseau, France; 3 School of Physical Science and Technology, Southwest University, Chongqing, 400715 China; 4 Center for High Technology Materials, University of New-Mexico, 1313 Goddard St SE, Albuquerque, NM 87106 USA; 5 Shanghai Engineering Research Center of Energy Efficient and Custom AI IC, ShanghaiTech University, Shanghai, 201210 China

**Keywords:** Semiconductor lasers, Nonlinear optics

## Abstract

Chaos in nonlinear dynamical systems is featured with irregular appearance and with high sensitivity to initial conditions. Near-infrared light chaos based on semiconductor lasers has been extensively studied and has enabled various applications. Here, we report a fully-developed hyperchaos in the mid-infrared regime, which is produced from interband cascade lasers subject to the external optical feedback. Lyapunov spectrum analysis demonstrates that the chaos exhibits three positive Lyapunov exponents. Particularly, the chaotic signal covers a broad frequency range up to the GHz level, which is two to three orders of magnitude broader than existed mid-infrared chaos solutions. The interband cascade lasers produce either periodic oscillations or low-frequency fluctuations before bifurcating to hyperchaos. This hyperchaos source is valuable for developing long-reach secure optical communication links and remote chaotic Lidar systems, taking advantage of the high-transmission windows of the atmosphere in the mid-infrared regime.

## Introduction

Chaos is a common phenomenon in numerous nonlinear dynamical systems, with features of random appearance and high sensitivity to initial conditions^1^. Since Haken’s theoretical prediction of chaos in laser systems in 1975^2^, chaotic oscillations have been observed in various types of lasers, including gas lasers^3–5^, solid-state lasers^6^, fiber lasers^7^, and semiconductor lasers^8^. Among these, semiconductor lasers are the most popular testbed owing to the high bandwidth, the compactness and the ease of control. Most semiconductor lasers belong to Class-B laser systems, where the carrier lifetime is much longer than the photon lifetime^8^. Consequently, the generation of chaos requires external perturbations, such as external optical or optoelectronic feedback, current or loss modulation, and optical injection^9^. However, chaos was also observed in free-running vertical-cavity surface-emitting lasers, owing to the nonlinear coupling between the two polarized modes in the vertical cavity^10^. In addition, quantum-dot micropillar lasers operated close to the quantum limit exhibited chaos as well^11,12^. The extensive and intensive investigations of chaos in semiconductor lasers have enabled various applications, including the chaotic secure communication^13^, the random number generation^14^, as well as the chaotic light detection and ranging (Lidar)^15^. In recent years, chaos is also applied in the field of optical reservoir computing, which advances the development of artificial intelligence^16–18^.

It is worthwhile to point out that most of the reported chaos of semiconductor lasers are operated in the near-infrared regime, especially in the O-band and the C-band communication windows of optical fibers. In contrast, mid-infrared chaos has potential applications in long-reach free-space secure optical (FSO) communication links and in remote chaotic Lidar systems, owing to the high-transmission windows (3–5 and 8–12 μm) of the atmosphere. In the 1980s, mid-infrared chaos has been demonstrated in gas lasers, such as CO_2_ lasers at 10.6 μm and He-Xe lasers at 3.5 μm^19,20^. However, the chaos bandwidth is limited up to the MHz range^21^. Recent work has focused on the quantum cascade lasers (QCLs) for the production of mid-infrared chaos^22–25^. For secure data transmission links, the utilization of hyperchaos with several positive Lyapunov exponents is highly desirable in contrast to chaos with only one positive Lyapunov exponent^26,27^. Recently, QCL’s hyperchaos was discovered and used to achieve the mid-infrared FSO communication based on the chaos synchronization technique^28^. This process successfully recovered a hidden message at a transmission rate of 0.5 Mbits/s with a few percent error that could be circumvented with the regular forward error correction. However, it is important to stress that the chaos bandwidth of QCLs is quite limited (MHz range), and particularly those lasers mostly produce low-frequency fluctuations (LFFs, also known as intermittent chaos) instead of hyperchaos^23,24,29,30^. This work reports fully-developed hyperchaos generated from mid-infrared interband cascade lasers (ICLs)^31,32^. Particularly, the mid-infrared chaotic signal reaches the GHz range for the first time, to the best of our knowledge. Most ICLs are grown on the GaSb substrate and emit in the spectral range of 3–6 μm, whereas InAs-based ICLs extend the lasing wavelength up to more than 10 μm^33–35^. The power consumption of ICLs is one or two orders of magnitude lower than the QCL counterpart^36^, and high-power ICLs have reached a power level of about 600 mW^37,38^. In contrast to QCLs, the stimulated emission of ICLs usually relies on the interband transition of type-II quantum wells, and the carrier lifetime is on the order of sub-nanosecond^39,40^. Therefore, ICLs are classified into Class-B laser systems like common quantum well lasers, and hence are more prone to produce fully-developed chaos. Here, we show the fully-developed hyperchaos of ICLs with the perturbation of external optical feedback. The chaos is proved to show a Lyapunov spectrum with three positive Lyapunov exponents, and the maximum Lyapunov exponent reaches about 2.1/ns. The electrical power spectrum is raised over a frequency span as broad as 2.0 GHz. Before producing the fully-developed chaos, the ICLs also generate periodic oscillations or LFFs, depending on the operation conditions.

## Results

### Laser device and experimental setup

**Fig. 1:**
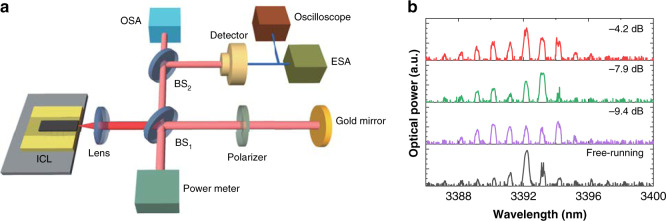
Experimental setup and laser emission spectra. **a** Experimental setup for the chaos generation in an ICL subject to external optical feedback. The feedback is provided by a gold mirror, and the feedback ratio is controlled by rotating the polarizer. OSA optical spectrum analyzer, ESA electrical spectrum analyzer, BS beam splitter. **b** The optical spectra of the ICL at 85 mA for several feedback ratios.

The ICL under study is a Fabry-Perot laser grown on the GaSb substrate by solid source molecular beam epitaxy (see Methods for device details). In order to trigger chaos, the ICL is perturbed by the external optical feedback. As shown in Fig. 1a, the optical feedback is provided by a gold mirror, and the feedback strength is adjusted through rotating the polarizer (see Methods for setup details). The ICL exhibits a lasing threshold of *I*_*th*_ = 78 mA, and saturates at 140 mA with a saturation power of 3.4 mW. The ICL has a single lateral mode at low pump currents, but high pump currents may excite higher-orders lateral modes. The linewidth broadening factor (LBF) of the ICL operated above threshold was measured to be around 2.2^41^. At the pump current of 85 mA, the laser emits several longitudinal modes around 3392 nm in Fig. 1b. When applying optical feedback, more longitudinal modes become lasing, due to the reduced lasing threshold^8^. At a feedback ratio of −4.2 dB, the threshold is reduced down to 73 mA. Although the chaos can broaden the spectral linewidth of the longitudinal modes, it is hard to be identified from the optical spectrum due to the limited resolution of the optical spectrum analyzer (0.1 nm). Based on the analysis of the optical spectrum, the dynamics of near-infrared laser diodes with optical feedback are usually classified into five regimes^42^. However, it is hardly possible to identify these five feedback regimes in ICLs, due to the resolution limitation of commercial mid-infrared spectrum measurement instruments. However, we believe that ICLs exhibit similar regimes as the near-infrared counterparts, because both lasers belong to the Class-B laser systems. On the other hand, the regime identification does not affect the demonstration of hyperchaos in this work.

### Chaos at a low pump current

**Fig. 2:**
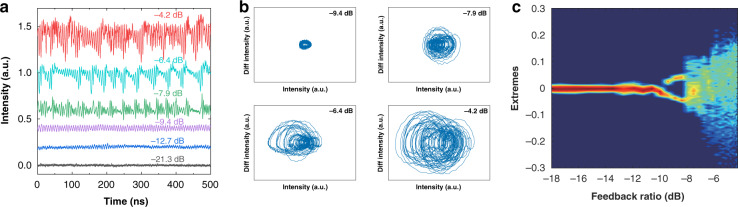
Evolution of time traces towards chaos at 85 mA. **a** Time traces and **b** phase portraits for various optical feedback levels. **c** Bifurcation diagram of the electrical power extremes.

When the ICL is pumped at a near-threshold pump current of 85 mA with an output power of 0.45 mW, Fig. 2a shows the evolution of the time traces with increasing feedback strength. The ICL produces continuous-wave output for weak feedback levels up to a feedback ratio of about −16 dB (example of –21.3 dB). For feedback ratios from −16 to −10 dB (example of −12.7 dB), weak oscillations arise in the time trace. This is because the optical feedback reduces the damping of the relaxation oscillation (RO) of the ICL^8^. Increasing the feedback ratios to the range of −10 to −8.0 dB (example of −9.4 dB), the ICL is destabilized and exhibits strong oscillations in the time trace. Both the time trace and the phase portrait in Fig. 2b prove that the dynamics is period-one (P1) oscillation, which shows a single period in the time series and one cycle in the phase portrait. The physical origin of P1 oscillations is that the relaxation oscillation is un-damped by the optical feedback through the Hopf bifurcation^43–46^. This is verified by the agreement of the P1 oscillation frequency with the RO frequency (see Section S1, Supplementary Information). P1 oscillations have been widely investigated in near-infrared laser diodes, which provide high-quality photonic microwaves for applications in radio-over-fiber communications. Most P1 oscillations are produced from lasers with optical injection^45–48^, whereas P1 oscillations from lasers with optical feedback have been studied as well^49–51^. When the feedback strength exceeds a critical feedback level, which quantifies the onset of chaos, the ICL starts to produce chaotic oscillations. The critical feedback level of the ICL is measured to be around −8.0 dB, which is more than 20 dB higher than that of common quantum well lasers^52,53^. However, this level is comparable to that of typical quantum-dot lasers^54,55^. The high critical feedback level of ICLs can be attributed to the small LBF and the large damping factor, as observed in our previous work^41,56,57^. Chaos for feedback ratios of −7.9, −6.4, and −4.2 dB in Fig. 2a exhibit typical irregular pulse oscillations. The corresponding phase portraits in Fig. 2b show that the chaotic oscillations become more and more complex with increasing feedback strength. The bifurcation diagram in Fig. 2c describes the power extremes (both maxima and minima) extracted from the time series. It clearly shows that the Hopf bifurcation point occurs around −10 dB, which is the onset of P1 oscillations with one maximum and one minimum. Beyond the critical feedback level of about −8.0 dB, the laser produces chaotic oscillations with multiple extremes. We did not observe intermediate dynamics between P1 oscillations and chaos in the measured ICL. However, common semiconductor lasers subject to optical feedback usually follow the quasi-periodic route to chaos^58^, although it is also possible to follow the period-doubling route under specific conditions^59^. The absence of intermediate dynamics in the tested ICL might be because that the corresponding regime is too small, and hence the dynamics is concealed by the multimode hopping and the optical noise. Simulations unveil that the ICL follows the typical quasi-periodic route to chaos like common laser diodes (see Section S2, Supplementary Information)^60,61^.

**Fig. 3:**
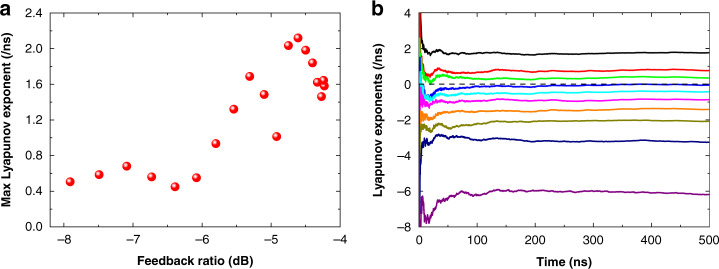
Analysis of the Lyapunov exponents at 85 mA. **a** Largest Lyapunov exponent as a function of the feedback ratio. **b** Lyapunov spectrum at the feedback ratio of −4.2 dB.

In order to quantify the sensitivity of chaos to the initial conditions, we extract the largest Lyapunov exponent from the time traces using Wolf’s algorithm^62,63^. A chaotic system at least has one positive Lyapunov exponent, which reflects the average exponential rate of divergence for nearby orbits in the phase space. This exponent also implies the time scale on which the system dynamics become unpredictable^21^. Figure 3a shows that the largest Lyapunov exponent is around 0.55/ns and has little change for the feedback ranging from −8.0 to −6.0 dB. With increasing feedback strength, it goes up to the maximum value of 2.1/ns at the feedback ratio of −4.6 dB. Further raising the feedback strength reduces the Lyapunov exponent down to 1.6/ns at the ratio of −4.2 dB. The reduction of the Lyapunov exponent can be attributed to the gain compression effect, since the strong optical feedback increases the output power^21^. It is stressed that these largest Lyapunov exponents are more than three orders of magnitude larger than those of LFFs in QCLs^23^. In order to characterize the dimensionality and the complexity of the chaos, we extract the Lyapunov spectrum using the method described in refs. ^64,65^. For the feedback ratio of −4.2 dB in Fig. 3b, the maximum five Lyapunov exponents are 1.76, 0.76, 0.34, −0.05, −0.43/ns, respectively. This spectrum clearly illustrates that the ICL exhibits a total number of three positive Lyapunov exponents. Therefore, the chaos is proved to be a fully-developed, high-dimensional chaos or a hyperchaos^21,26,27^. It is remarked that the largest Lyapunov exponent at −4.2 dB in Fig. 3a is similar but slightly different to that in Fig. 3b, which is due to the different calculation methods. Detailed embedding parameters for the extraction of the largest Lyapunov exponents and the Lyapunov spectra are listed in Section S3, Supplementary Information.

**Fig. 4:**
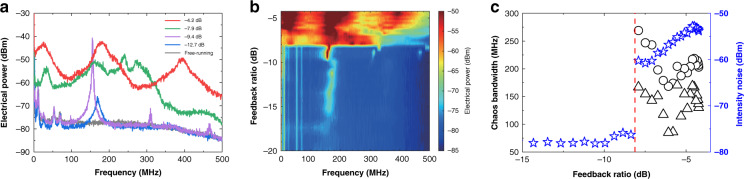
Evolution of electrical spectra towards chaos at 85 mA. **a** Electrical spectra for various feedback levels. **b** Electrical power distribution map as functions of feedback ratio and Fourier frequency. **c** Chaos bandwidth with Definition I (Circles) and Definition II (triangles). Stars stand for the averaged intensity noise within the photodetector bandwidth. The dashed line indicates the critical feedback level.

Figure 4 shows the characteristics of the electrical spectrum of the ICL with optical feedback. In Fig. 4a, the free-running ICL shows a smooth electrical spectrum except the low-frequency part. Below 100 MHz, the noisy spikes are mainly due to the mode partition noise as well as the technical noise sources including the current source noise, the thermal noise, and the mechanical noise^66,67^. The spectrum does not show any RO peak, suggesting that the ICL is strongly damped. This observation is consistent with the modulation responses^68–70^, where no resonance peak appears either. Our recent work quantitatively proved that the K factor of an ICL is as large as 31.4 ns, and the strong damping effect arises from the high gain compression factor of 5.1 × 10^−15^ cm^3^^57^. The strong damping effect also leads to the absence of the resonance peak in the relative intensity noise of ICLs, which prevents the observation and the extraction of the RO frequency in our previous work^67^. Consequently, ICLs resemble quantum-dot lasers, where the ROs are usually overdamped as well^71,72^. When the optical feedback with a feedback ratio of −12.7 dB is applied to the ICL, a small peak appears around 168 MHz, which determines the oscillation period of the corresponding time trace in Fig. 2a. The peak frequency is much smaller than the external cavity frequency of 417 MHz, and thus the peak must be due to the underdamped RO. This oscillation frequency has been demonstrated to swing around the RO frequency of the free-running laser^8,73^. Therefore, we can deduce that the RO frequency of the free-running ICL is roughly around 168 MHz, although this evaluation is not highly accurate. Increasing the feedback level to −9.4 dB, the ICL exhibits a typical P1 oscillation at 155 MHz, and the oscillation peak amplitude is about 40 dB higher than the background noise level. Detailed relations between the RO frequency and the P1 oscillation frequency are discussed in Section S1, Supplementary Information. At feedback ratios of −7.9 and −4.2 dB, the ICL produces chaotic oscillations and the electrical power levels are substantially raised over a broad frequency range, up to the bandwidth limit (450 MHz) of the photodetector. The map in Fig. 4b displays the evolution of the electrical power distribution as functions of the Fourier frequency and the feedback level. It is shown that the frequency of the weak RO slightly increases with increasing feedback level from −16 to −10 dB. This is because the RO frequency is proportional to the square root of optical power, which is raised by the optical feedback^73,74^. In addition, it is known that the RO frequency also varies with the feedback length and the feedback phase^75,76^. At the onset of P1 oscillation around −10 dB, the oscillation frequency abruptly shifts to a slightly smaller value. Beyond the critical feedback level of −8.0 dB, the ICL exhibits chaotic oscillations within a broad feedback level window, up to the feedback limit (−4.2 dB) of the experimental configuration. In order to quantify the bandwidth of the chaotic signals, we employ Definition I that the frequency span from DC to the cutoff frequency, which contains 80% of the total power in the electrical spectrum^77^. Using this definition, Fig. 4c demonstrates that the chaos bandwidth (circles) firstly declines and then rises with the increasing feedback ratio. The maximum chaotic bandwidth is 269 MHz, which is reached right above the critical feedback level. It is remarked that this chaos bandwidth is almost two orders of magnitude broader than the LFF bandwidth of QCLs^22,28,78^. Figure 4c also plots the chaos bandwidth (triangles) using Definition II that the sum of discrete spectral segments accounting for 80% of the total power in the electrical spectrum^79^. It is shown that the chaos bandwidth of Definition II is smaller than that of Definition I, whereas both exhibit similar evolution trend versus the feedback strength. In addition, Fig. 4c shows that the chaos abruptly raises the intensity noise (stars) by more than 15 dB at the critical feedback level, and the noise level continuously rises with increasing feedback ratio.

### Chaos at a high pump current

**Fig. 5:**
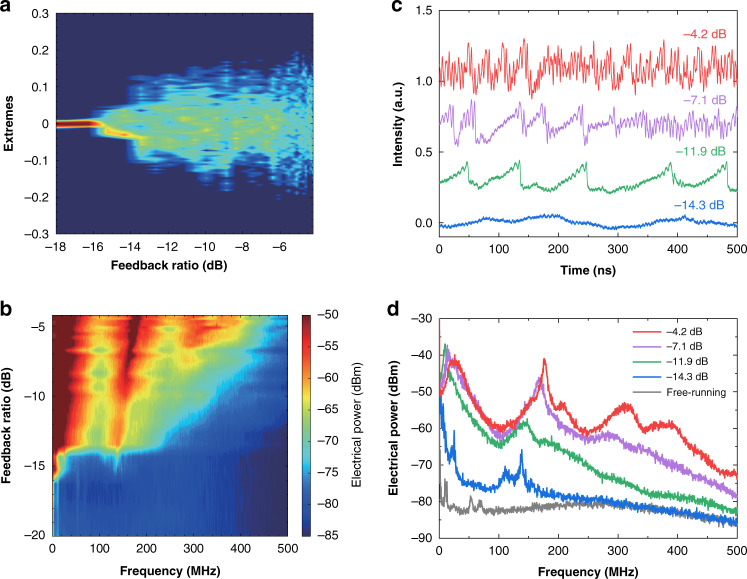
Chaos at a high pump current of 105 mA. **a** Bifurcation diagram and **b** electrical power distribution towards chaos. Examples of **c** time traces and **d** electrical spectra for several feedback ratios.

The ICL produces chaos not only at near-threshold pump currents, but also at high ones. This is in contrast to QCLs, where the chaotic LFFs mostly occur at near-threshold currents^22–25^. Our previous work has shown that QCLs operated far above the threshold were much more stable^80,81^. When the ICL is pumped at 105 mA with an output power of 1.7 mW, both the bifurcation diagram in Fig. 5a and the electric power distribution map in Fig. 5b show that the ICL does not exhibit any periodic oscillations. Instead, the ICL produces LFFs before bifurcating to the regime of fully-developed chaos. Consequently, the ICL follows the LFF route to chaos^29,30,82^. The appearance of LFFs is likely due to the fact that the ICL exhibits more longitudinal modes at a higher pump current, which is worthwhile of theoretical studies in future work^83,84^. For feedback levels ranging from −16 to −14 dB (example of −14.3 dB) in Fig. 5d, the multimode hopping slightly raises the noise level at frequencies below 200 MHz, which leads to the weak fluctuations in the corresponding time trace in Fig. 5c. The ICL produces LFFs for feedback ratios of −14 to −8.0 dB (example of −11.9 dB). The LFFs in Fig. 5c show irregular power jump-ups with gradual power increase and drastic power decrease. This is in contrast to typical LFFs observed in common laser diodes, which are featured with random power dropouts with sudden power decrease and gradual power recovery^29,30^. However, LFFs with power jump-ups have been indeed observed in semiconductor lasers biased well above threshold^85^. In addition, we also observe coexistence of power jump-ups and dropouts in the experiment. The LFF leads to the power enhancement of the electrical spectra in Fig. 5b, d, and the bandwidth broadens with rising feedback level. Besides, a dominated peak appears around the RO frequency, and the peak frequency increases with the feedback level as well. Further increasing the feedback level to the range from −8.0 to −6.0 dB (example of −7.1 dB), both the LFF and the chaos coexist in the time trace of Fig. 5c, and the bandwidth of the power spectrum in Fig. 5d further broadens. The dynamics finally evolves into fully-developed chaos when the feedback level surpasses −6.0 dB, which is 2.0 dB higher than the onset of fully-developed chaos at the low pump current. Exampled fully-developed chaos at −4.2 dB is displayed in Fig. 5c. Detailed characteristics of the LFFs and the chaos are discussed in Section S4, Supplementary Information. Lastly, it is remarked that the chaos generations at 85 and 105 mA are very stable. Besides, the ICL stably produces chaos for pump currents ranging from near threshold up to about 130 mA (1.7 × *I*_*th*_).

### Chaos of a distributed feedback ICL

**Fig. 6:**
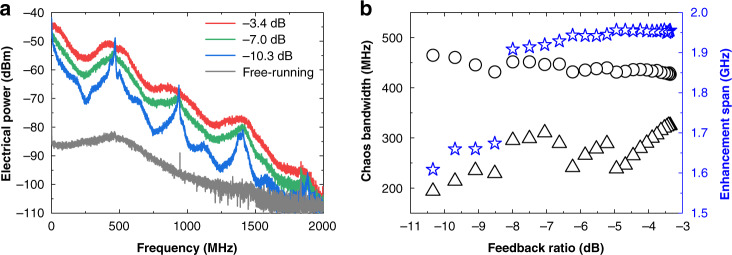
Chaos of the DFB ICL. **a** Electrical spectra at several feedback ratios. **b** Chaos bandwidth with Definition I (Circles) and Definition II (triangles). Stars stand for the power enhancement span. The feedback length is 30 cm.

In order to verify that the generation of chaos is a universal phenomenon in ICLs rather than in a peculiar Fabry–Perot ICL device, we tested a commercial single-mode distributed feedback (DFB) ICL (from Nanoplus)^86^. The ICL shows a lasing threshold of *I*_*th*_ = 23 mA and emits on a single mode around 3.38 μm. The above-threshold LBF is measured to be around 2.3. For an external cavity length of 30 cm, we find that the DFB ICL stably produces chaos for pump currents ranging from near threshold up to 50 mA (2.2 × *I*_*th*_). When the ICL is pumped at 30 mA with an output power of 1.4 mW, the ICL produces fully-developed chaos beyond the critical feedback level of −10.3 dB, and the electrical spectra are shown in Fig. 6a. The chaos bandwidth with Definition I (circles) in Fig. 6b generally decreases from 465 MHz at the feedback ratio of −10.3 dB down to 427 MHz at −3.4 dB. In contrast, the bandwidth with Definition II (triangles) generally increases from 194 MHz up to 325 MHz. Meanwhile, the electrical power spectrum is raised over a frequency span from 1.61 GHz up to 1.96 GHz.

**Fig. 7:**
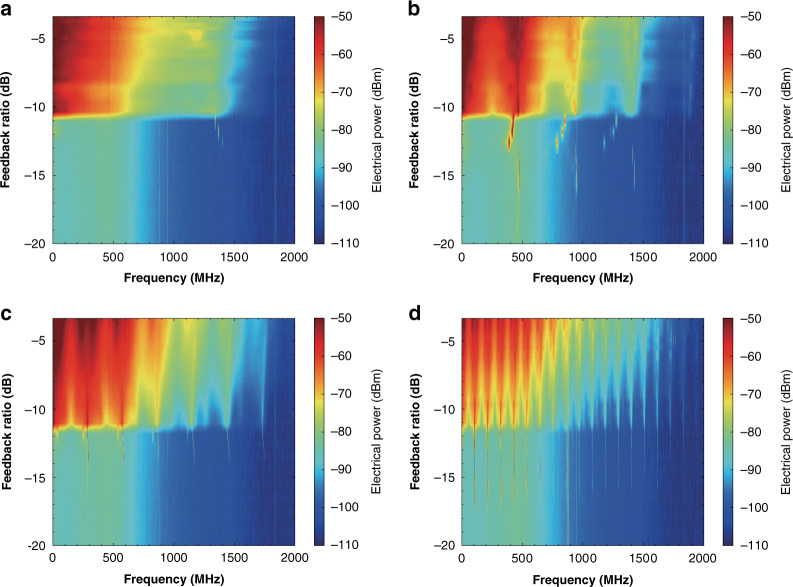
Chaos of the DFB ICL for different feedback lengths. Electrical power distribution for feedback lengths of **a** 10 cm, **b** 30 cm, **c** 50 cm, and **d** 136 cm.

It is known that the feedback dynamics of semiconductor lasers are not only determined by the pump current and the feedback strength, but also by the feedback cavity length. Generally, the critical feedback level for the onset of chaos decreases with increasing feedback length^56^. Most near-infrared laser diodes with optical feedback from a fiber loop are operated in the long-cavity regime, where the external cavity frequency is much smaller than the RO frequency. On the other hand, laser diodes with on-chip feedback are operated in the short-cavity regime, which is compact and desirable for practical applications^87,88^. For the ICL with optical feedback using free-space optical components, nevertheless, it is challenging to reach the long-cavity regime due to the divergence of the Gaussian laser beam. Our experimental setup can achieve a minimum feedback length of 10 cm and a maximum one of 136 cm. Figure 7 shows that the DFB ICL produces chaos for all the feedback lengths of 10, 30, 50, and 136 cm. The critical feedback levels fall in the range of −10 to −11 dB, and do not show a clear declining trend with increasing feedback length. This is attributed to the reduced coupling ratio of the feedback light into the laser chip for the long external cavity. Before the appearance of chaos, the ICL generates periodic oscillations around the external cavity frequency for all the feedback lengths. In addition, the chaos spectra in Fig. 7 show obvious peaks at multiples of the external cavity frequency. Interestingly, the DFB ICL with optical feedback does not show apparent signature of RO, which might be due to the extremely strong damping effect. As a result, the optical feedback can not un-damp the RO like in QCLs, which requires further investigations in future work^78,89^. Effects of the feedback length on the chaos bandwidth are discussed in Section S5, Supplementary Information.

## Discussion

The chaos bandwidths of the two tested ICLs are smaller than the common GHz bandwidth of near-infrared laser diodes^79^. This is because the chaos bandwidth is roughly determined by the RO frequency of semiconductor lasers. However, the tested ICL samples are not designed for high-speed operations. Reported modulation bandwidths of ICLs include 120 MHz^57^, less than 200^90^, 850^69^, 3.2 GHz^68^, and the record value of 5.0 GHz^70^. These bandwidths are much smaller than the modulation bandwidths of high-speed near-infrared laser diodes^91,92^. The record bandwidth determined by the RO frequency (more than 40 GHz) has reached 60 GHz, while the photon-photon resonance has lifted the modulation bandwidth up to 108 GHz^92^. The bandwidth of ICLs is likely to be limited by the carrier transport process through the thick separate confinement layer and the thick active region, as well as the strong gain compression effect (see Section S2, Supplementary Information)^57,66^. However, the underlying physical mechanisms are not fully understood yet. It is not surprising that the chaos bandwidth of ICLs can be enhanced to the GHz level in the future by reducing the carrier transport time and/or by limiting the gain compression effect^66^. On the other hand, it will be challenging to detect such a broadband chaos, because the bandwidth of commercial HgCdTe photodetector is limited to be around 1.0 GHz. In this case, advanced high-speed photodetectors such as interband cascade or quantum well infrared photodetectors have to be employed^93,94^.

In summary, we have demonstrated the fully-developed hyperchaos generation from mid-infrared ICLs, which was triggered by the external optical feedback. The chaos shows a Lyapunov spectrum with three positive Lyapunov exponents. The largest Lyapunov exponent reaches up to 2.1/ns. The chaos bandwidth is as broad as 465 MHz and the electrical spectrum is raised over a frequency range of 2.0 GHz. The ICLs do not only produce hyperchaos at near-threshold currents but also at high ones. Before bifurcating to chaos, the ICL exhibits periodic oscillations when operated close to the threshold, and exhibits LFFs when operated well above the threshold. Although the chaos is demonstrated with low-power ICLs, we believe that the chaos of high-power ICLs can be used to develop long-reach secure FSO communication links and remote chaotic Lidar systems.

## Methods

The ICL under study consists of 7 cascading gain stages, which are formed by W-shape InAs/GaInSb type-II quantum wells. The laser has a ridge width of 9.0 μm and a cavity length of 1.5 mm. Both laser facets are as-cleaved without any coatings. The ICL is epilayer-up mounted, and is covered with gold by electroplating to improve the thermal dissipation. The ICL is mounted on a heat sink and its temperature is maintained at 20 °C by using a thermo-electric controller. The pump current is supplied by a low-noise battery current source (LDX-3620B). As shown in Fig. 1a, the laser output is collimated by an aspherical lens with a focal length of 4.0 mm. The light is split into two paths by a beam splitter (BS_1_). One path provides the optical feedback through a gold mirror, which is placed 36 cm away from the laser sample. The feedback strength is adjusted by rotating the polarizer, and the feedback power is monitored by a power meter. The optical feedback effect is dominated by the transverse-electric (TE) component, while the transverse-magnetic (TM) one is negligible. This is because the gain of ICL is dominated by the TE gain, and hence the laser is TE polarized^95,96^. On the other hand, the TM component of the optical feedback can be completely removed by inserting another polarizer aligned at the TE direction right after BS_1_. The feedback ratio is defined as the ratio of the mirror reflected power to the laser output power. The maximum feedback ratio offered by this configuration reaches up to −4.2 dB. The other optical path is used for characterization. The optical spectra are measured by a grating-based optical spectrum analyzer (Yokogawa AQ6376) with a resolution of 0.1 nm. The optical signal is converted to the electrical one by a HgCdTe photodetector (Vigo PVI-4TE-6) with a nominal bandwidth of 450 MHz. The electrical spectra are measured by a broad bandwidth electrical spectrum analyzer, and the time series are recorded on a high-speed oscilloscope.

## Supplementary information

Supplemental information for the main manuscript

## Data Availability

The data that support the findings of this study are available from the corresponding author upon reasonable request.
