# Supplementary material for: Mid-infrared hyperchaos of interband cascade lasers
Source: Light Sci Appl. 2022 Jan 2;11:7. doi: 10.1038/s41377-021-00697-1 (PMC8720313; doi:10.1038/s41377-021-00697-1)
Supplement: Supplementary file 1 — Supplemental information for the main manuscript [file 41377_2021_697_MOESM1_ESM.docx]

**Supplementary Information**

**Mid-infrared hyperchaos of interband cascade lasers**

Yu Deng, Zhuo-Fei Fan, Bin-Bin Zhao, Xing-Guang Wang, Shiyuan Zhao, Jiagui Wu^*^, Frédéric Grillot^*^, and Cheng Wang^*^

*Correspondence: Cheng Wang (wangcheng1@shanghaitech.edu.cn); Frédéric Grillot (frederic.grillot@telecom-paris.fr); Jiagui Wu (mgh@swu.edu.cn)

**Section S1: Relaxation oscillation frequency and period-one oscillation frequency**

Because the ICLs studied in the main manuscript do not support the direct current modulation, we fabricated another Fabry-Perot ICL with radio-frequency package for the investigation of the modulation response. It is found that the modulation response of the ICL does not show any resonance peak due to the strong damping effect. The maximum modulation bandwidth is around 120 MHz, associated with an intrinsic bandwidth of about 180 MHz. The K factor is 31.4 ns, the differential gain is 7.8×10^−16^ cm^2^, and the gain compression factor is as high as 5.1×10^−15^ cm^3^. Detailed characteristics of the modulation response refer to [S1]. The electrical spectrum in Fig. S1(a) shows that the weak optical feedback excites underdamped relaxation oscillation (RO), with a low and broad peak (lower panel). On the other hand, moderate optical feedback stimulates the period-one (P1) oscillation, with a high and sharp peak (upper panel). It is demonstrated that the P1 oscillation frequencies meet well with the underdamped RO frequencies for all the studied currents of 79, 81, and 84 mA. Based on the analysis of the modulation response in [S1], we extract the overdamped RO frequency of the free-running ICL (squares) in Fig. S1(b). The RO frequency rises with the increasing pump current. Meanwhile, both the underdamped RO frequency with optical feedback (circles) and the P1 oscillation frequency (stars) vary around the overdamped RO frequency of the free-running ICL. This observation is in agreement with that of near-infrared laser diodes [S2]. Consequently, we conclude that both the underdamped RO with optical feedback and the P1 oscillation arise from the overdamped RO of ICLs, which is similar to common laser diodes.


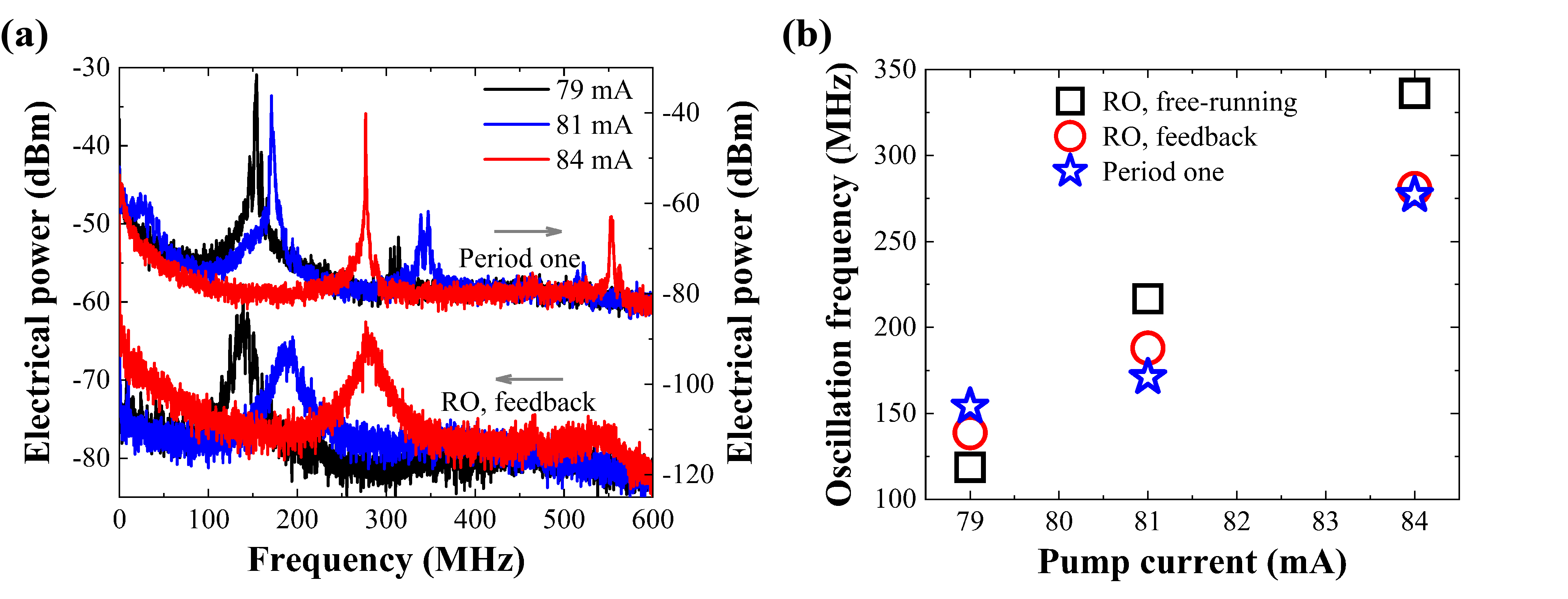


**Fig. S1** (a) Electrical spectrum of the ICL with optical feedback for several pump currents. Upper panel shows period-one oscillations, lower panel shows RO with optical feedback. (b) RO frequency of the free-running laser (squares) and of the laser with weak feedback (circles), as well as the P1 oscillation frequency (stars). The lasing threshold of the free-running ICL is *I_th_* =75 mA.

**Section S2: Rate equation modeling**

The dynamics of the carrier (*N*), the photon (*S*) and the phase (*ϕ*) of the electric field for the ICL with external optical feedback are described by [S3]

$\frac{dN}{dt}=\eta\frac{I}{q}-\Gamma_{p}v_{g}g_{0}S-\frac{N}{\tau_{sp}}-\frac{N}{\tau_{aug}}$ (S1)

 (S2)

 (S3)

where *I* is the pump current, *η* is the current injection efficiency, and *q* is the elementary charge. *τ_sp_* is the spontaneous emission lifetime (15 ns) [S4], *τ_aug_* is the Auger recombination lifetime (1.08 ns) [S4], and *τ_p_* is the photon lifetime in the cavity (10 ps). *g_0_* is the material gain without including the gain compression effect, *v_g_* is the group velocity of light (7.8 cm/ns), *Г_p_* is the optical confinement factor (0.04), *m* is the number of cascading gain stages (7), *α_H_* is the linewidth broadening factor (2.2), and *β* is the spontaneous emission factor (10^−4^), and other simulation parameters are listed in [S3]. The optical feedback effect is characterized by the classical Lang-Kobayashi model [S5]. *r_ext_* is the feedback ratio, *τ_ext_* is the feedback delay time (2.4 ns), *k_c_* is the coupling coefficient of the feedback light into the laser cavity. The phase difference Δ*ϕ* is given by Δ*ϕ*=*ϕ*_0_*+ϕ*(*t*)−*ϕ*(*t*−*τ_ext_*), with *ϕ*_0_ being the initial feedback phase. *ϕ*_0_ is expressed as *ϕ*_0_= *ω*_0_×*τ_ext_,* with *ω*_0_ being the lasing frequency of the free-running laser. Because the initial phase is very sensitive to the vibration of the feedback delay length, we treat it as a free parameter, which is fixed at zero in the following simulations. In order to remove the impacts of multimode emissions and the spontaneous emission noise on the bifurcation diagram, which can interfere the identification of the route to chaos, both processes are not included in the rate equation model.

Figure S2 illustrates the bifurcation routes to chaos for different operation conditions. For *I*=1.5×*I_th_* and *τ_ext_*=2.4 ns in Fig. S2(a), the ICL exhibits a small regime of period-one oscillations (−25.6 dB to −24.4 dB), similar as the experimental observation in Fig. 2 of the main manuscript. It is shown that the simulated ICL produces intermediate dynamics of quasi-periodic oscillations in the narrow regime from −24.2 dB to −23.0 dB. However, this quasi-periodic regime is concealed by the multimode emission and the spontaneous emission in the experiment. Enhancing the pump current to *I*=3.0×*I_th_* in Fig. S2(b), the period-one oscillation regime significantly shrinks to a very narrow window (−18.0 dB to −17.8 dB), and the dynamics almost directly evolves from the continuous-wave state to the chaotic state, similar as the experimental results in Fig. 5 of the main manuscript. On the other hand, both the period-one oscillation regime and the quasi-periodic oscillation regime can be broadened either by reducing the pump current (*I*=1.1×*I_th_* in Fig. S2(c)) or by shortening the feedback delay time (*τ_ext_*=1.0 ns in Fig. S2(d)) [S6,S7], which are helpful for the identification of the detailed route to chaos in future work.


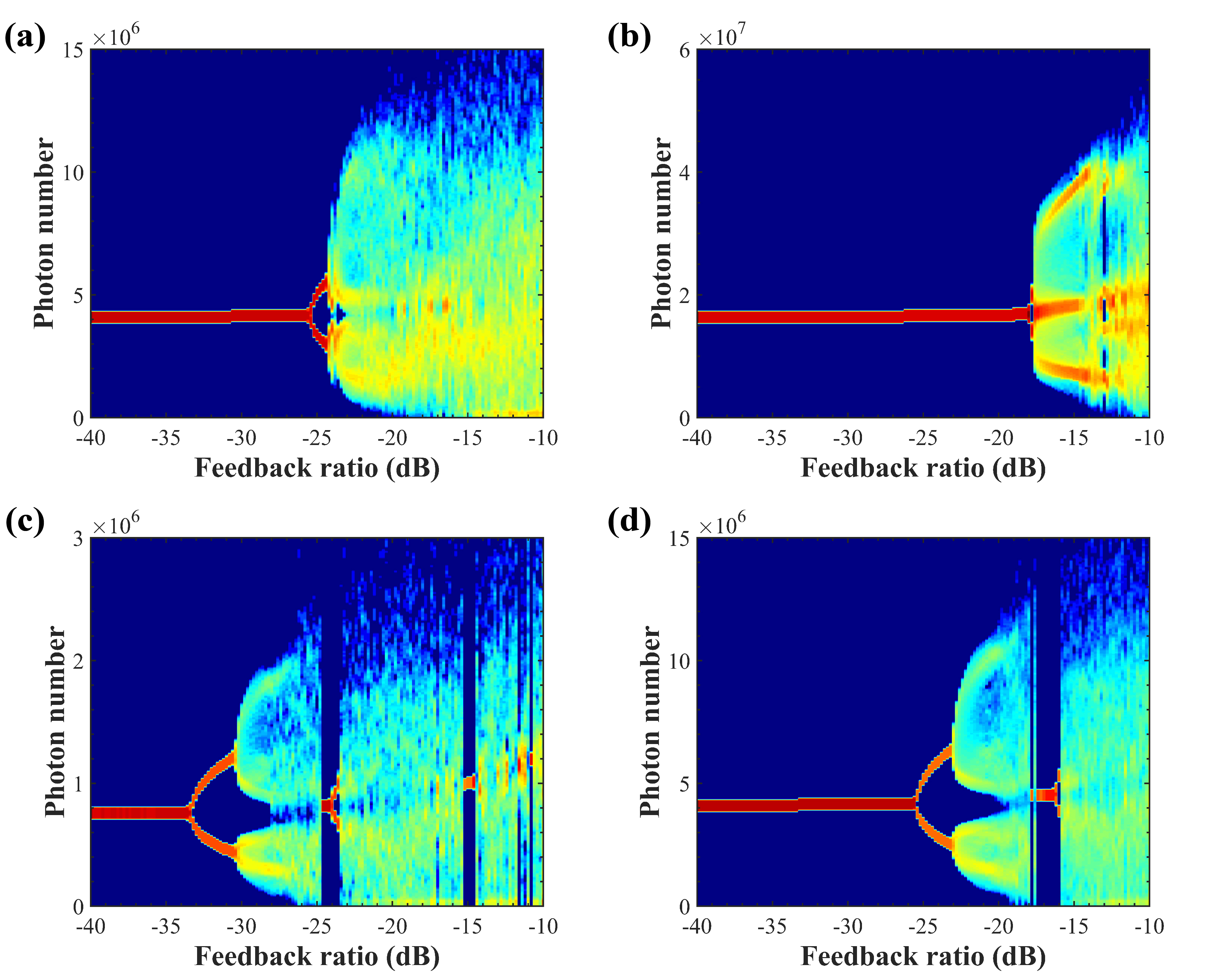


**Fig. S2** Simulated bifurcation diagrams for various operation conditions. (a) Pump current is 1.5×*I_th_* and time delay is 2.4 ns. (b) Pump current is 3.0×*I_th_* and time delay is 2.4 ns. (c) Pump current is 1.1×*I_th_* and time delay is 2.4 ns. (d) Pump current is 1.5×*I_th_* and time delay is 1.0 ns. The initial feedback phase is fixed at zero. The lasing threshold current is *I_th_*=29 mA.


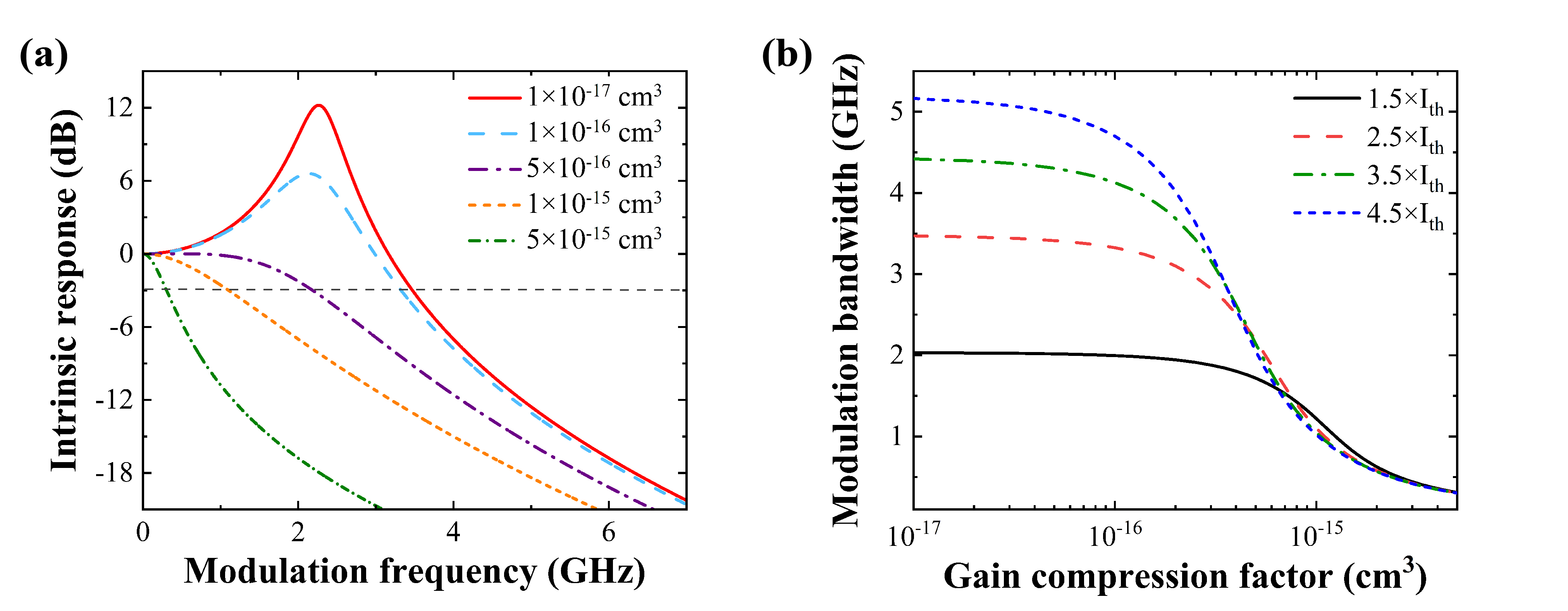


**Fig. S3** Simulated modulation response of the free-running ICL. (a) Modulation responses for various gain compression factors at the pump current 2.5×*I_th_.* (b) 3-dB modulation bandwidth as a function of the gain compression factor for various pump currents.

Based on Eqs. (S1)-(S3), one can take into account the gain compression effect by replacing the gain term *g_0_* with g=*g_0_*/(1+*εS*), as well as the carrier transport process through the separate confinement hetero-structure (details will be reported elsewhere). It is known that the gain compression effect limits the maximum modulation bandwidth *f_3dB,max_* of free-running semiconductor lasers through the following formulas [S8]:

$f_{3dB,\max}=2\sqrt{2}\pi/K$ (S4)

$K=4\pi^{2}\tau_{p}\left[1+\frac{\Gamma_{p}\varepsilon g}{a(1+\varepsilon S)}\right]$ (S5)

where *K* is the K factor and *a* is the differential gain including the gain compression effect. Figure S3(a) shows the simulated modulation responses of a free-running ICL for various gain compression factors. It is proved that a large gain compression factor indeed leads to the absence of the resonance peak, as experimentally observed in [S1]. Meanwhile, the modulation bandwidth is reduced as well. Figure S3(b) presents that the modulation bandwidth declines nonlinearly with increasing gain compression factor for various pump currents. At the pump current of 4.5×*I_th_*, the modulation bandwidth decreases from 5.2 GHz for the gain compression factor of 1.0×10^-17^ cm^3^ down to 0.3 GHz for the factor of 5.0×10^-15^ cm^3^. This simulated bandwidth range meets well with the reported modulation bandwidths in experiment, as described in the main manuscript.

**Section S3: Lyapunov exponents**

For the extraction of the largest Lyapunov exponent in Fig. 3(a), the embedding parameters are described in [S9], and the parameter values are carefully selected and listed in Table S1. For the extraction of the Lyapunov spectrum in Fig. 3(b), the embedding parameters are described in [S10,S11], and the parameter values are carefully selected and listed in Table S2.

**Table S1** Embedding parameters for the largest Lyapunov exponent

| Time span | Sampling rate | Time delay | Dimension | Grid resolution | Evolution time | Minimum separation at replacement | Maximum separation  at replacement |
| --- | --- | --- | --- | --- | --- | --- | --- |
| 1000 ns | 20 Gsa/s | 2 ns | 3 | 10 | 1 ns | 0.01 | 0.06 |

**Table S2** Embedding parameters for the Lyapunov spectrum

| Time span | Sampling rate | Time delay | Evolution time | Local embedding dimension | Local and global dimensions |
| --- | --- | --- | --- | --- | --- |
| 500 ns | 20 Gsa/s | 2 ns | 1 ns | 10 | 30 |

**Section S4: Chaos at the high pump current**

The low frequency fluctuations (LFFs) in semiconductor lasers are featured with typical statistical characteristics. That is, the probability distribution of the time interval between the power dropouts or jump-ups of the LFFs exhibits a zero probability (dead zone) at short time intervals, followed by a rise to the maximum probability at a certain interval, and then an exponential decay for long intervals [S12]. In the main manuscript of Fig. 5, the ICL pumped at the high current of 1.35×*I_th_* exhibits LFFs for feedback ratios in the range of −14 dB to −8.0 dB. As an example, Fig. S4(a) displays the statistical distribution of the LFF at the feedback ratio of −11.9 dB. The LFF indeed shows a dead zone for short time intervals up to 40 ns. Then, the probability climbs up to the peak value at a time interval of 120 ns. The distribution probability has a long tail for large time intervals, and it declines with the typical exponential trend. The mean time interval of the power dropout events is around 124 ns. In addition, it is found that the mean time interval decreases with increasing feedback strength (not shown). Figure S4(b) shows that the LFF bandwidth with Definition I (circles) rises with increasing feedback level. At the onset of chaos with coexisted LFF (−8.0 dB), the bandwidth abruptly broadens from 43 MHz to 115 MHz. The fully developed chaos emerges at −6.0 dB, and the chaos bandwidth continuously goes up to about 180 MHz for the feedback ratio of −4.2 dB. Interestingly, the LFF bandwidth with Definition II (triangles) is almost the same as that with Definition I. However, the chaos bandwidth with Definition II is generally smaller than the latter. The averaged intensity noise (stars) has a substantial increase at the onset of LFF. Interestingly, the averaged noise increases almost linearly with the feedback strength up to the maximum feedback ratio of −4.2 dB. Figure S4(c) shows that the largest Lyapunov exponent increases slightly with the feedback ratio both in the regime of LFF and in the regime of coexisted LFF and chaos. Once the dynamics evolves into the fully-developed chaos regime, the Lyapunov exponent rises rapidly to the maximum value of 1.4 /ns at the feedback ratio of −4.2 dB. In comparison with the chaos produced close to the threshold (see Figs. 2–4 in the main manuscript), the chaos generated well above threshold has a narrower bandwidth and a smaller Lyapunov exponent. Figure S4(d) shows the Lyapunov spectrum for the feedback ratio of −4.2 dB. The maximum five Lyapunov exponents are 1.35, 0.49, 0.01, −0.41 and −0.89 /ns, respectively. Therefore, the spectrum includes a total number of two positive Lyapunov exponents, which proves that the chaos is a high-dimensional one as well. This high current has one less positive Lyapunov exponent than the low current in Fig. 3(b), which might be due to the strong gain compression effect [S1,S13]. The hyperchaos at high pump currents is favorable for practical applications, which require both complex chaotic dynamics and high chaos power. It is remarked that the ICL operated at a high pump current may produce multiple lateral modes, and the interaction of the modes may complicate the nonlinear dynamics including the chaos behavior [S13].


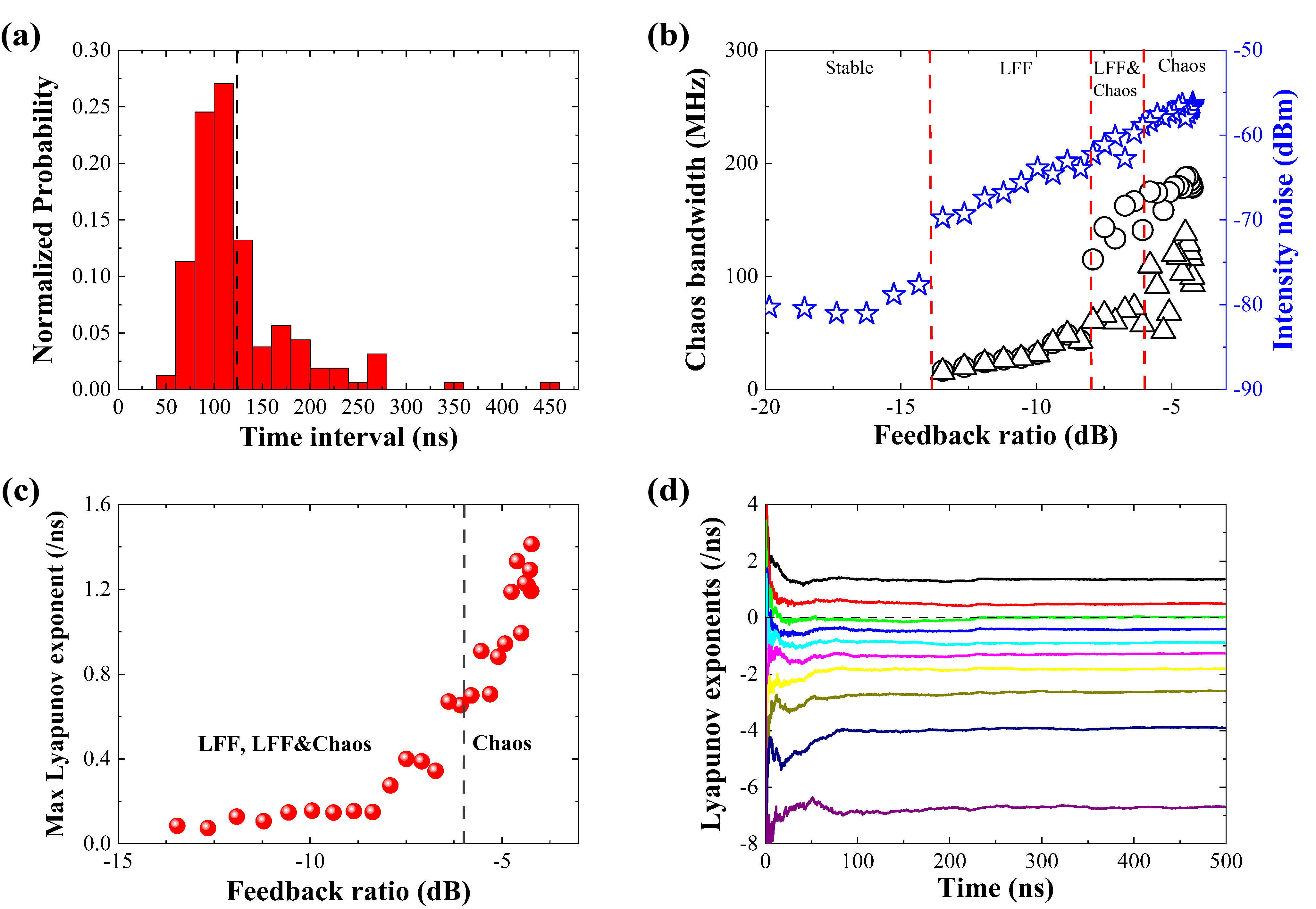


**Fig. S4** (a) Statistical distribution of the time interval between the power dropouts of the LFF at the feedback ratio of −11.9 dB. The dashed line indicates the averaged time interval. (b) Chaos bandwidth with Definition I (circles) and Definition II (triangles). Stars stand for the averaged intensity noise within the photodetector bandwidth. The dashed lines separate the regimes of continuous wave, LFF, coexistence of LFF-chaos, and chaos. (c) Largest Lyapunov exponent as a function of the feedback ratio. The dashed line represents the onset of fully-developed chaos. (d) Lyapunov spectrum at the feedback ratio of −4.2 dB.

**Section S5: Chaos of the distributed feedback ICL**

Figure S5 shows the chaos bandwidth of the distributed feedback ICL for several feedback lengths. With the bandwidth Definition I, Fig. S5(a) shows that the shortest feedback length of 10 cm excites the smallest chaos bandwidth. On the other hand, the maximum chaos bandwidth is achieved at 30 cm and 136 cm. With the bandwidth Definition II, Fig. S5(b) shows that the chaos bandwidth generally broadens with increasing feedback length. Therefore, the minimum chaos bandwidth is achieved at the feedback length of 10 cm, and the maximum one is at 136 cm. The dependence of the chaos bandwidth on the feedback length is due to the effect that the RO frequency is varied by the feedback length [S2,S14]. In the long-cavity regime (where the external cavity frequency is much smaller than the RO frequency), nevertheless, the chaos bandwidth of common laser diodes is almost independent on the feedback length [S15].


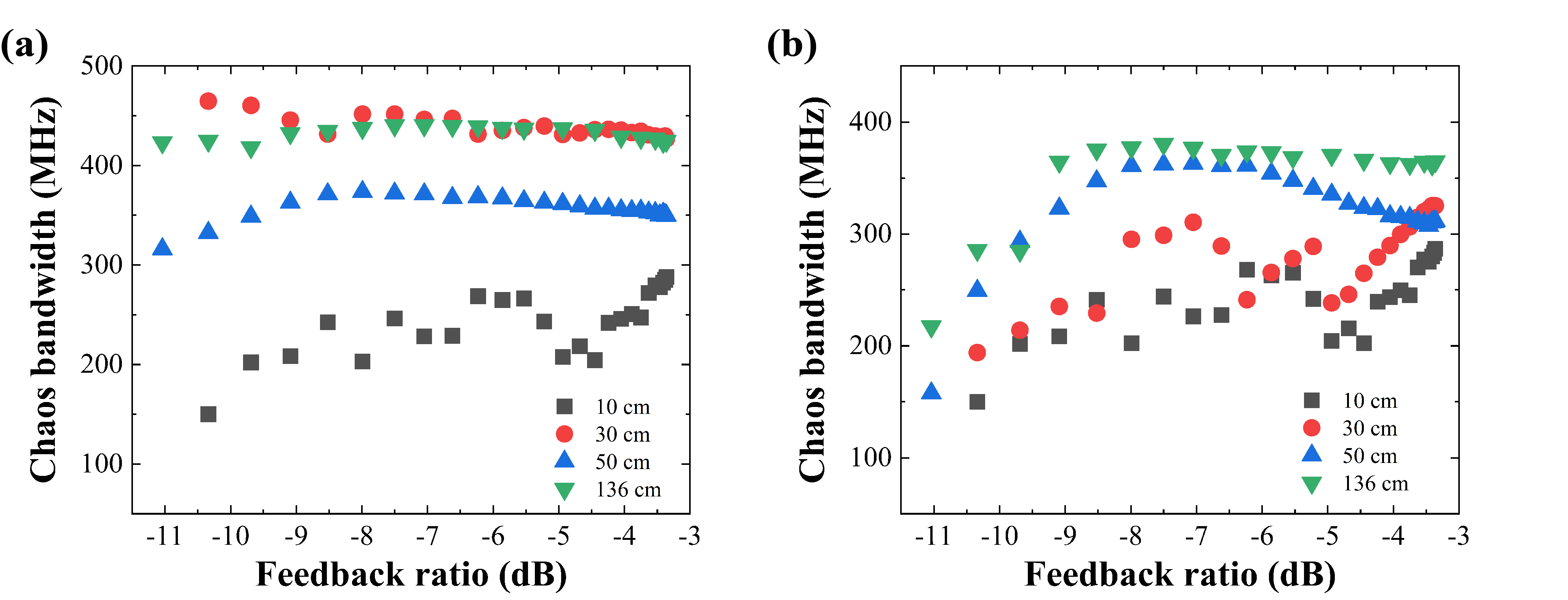


**Fig. S5** Effects of the feedback length on the chaos bandwidth. (a) Bandwidth with Definition I. (b) Bandwidth with Definition II.

**References**

1. Fan, Z. F., Deng, Y., Ning, C., Liu, S. M. & Wang, C. Differential gain and gain compression of an overdamped interband cascade laser. *Appl. Phys. Lett.* **119**, 081101 (2021).
2. Ye, S. Y. & Ohtsubo, J. Experimental investigation of stability enhancement in semiconductor lasers with optical feedback. *Opt. Rev.* **5**, 280–284 (1998).
3. Deng, Y. & Wang, C. Rate equation modeling of interband cascade lasers on modulation and noise dynamics. *IEEE J. Quantum Electron.* **56**, 2300109 (2020).
4. Bewley, W. W. et al. Lifetimes and Auger coefficients in type-II W interband cascade lasers. *Appl. Phys. Lett.* **93**, 041118 (2008).
5. Lang, R. & Kobayashi, K. External optical feedback effects on semiconductor injection laser properties. *IEEE J. Quantum Electron.* **16**, 347–355 (1980).
6. Ruiz-Oliveras, F. R. & Pisarchi, A. N. Phase-locking phenomenon in a semiconductor laser with external cavities. *Opt. Express* **14**, 12859–12867 (2006).
7. Kim, B., Locquet, A., Li, N., Choi, D. & Citrin, D. S. Bifurcation-cascade diagrams of an external-cavity semiconductor lasers: experiment and theory. *IEEE J. Quantum Electron.* **50**, 965–971 (2014).
8. Coldren, L. A., Corzine, S. W. & Masanovic, M. L. *Diode Lasers and Photonic Integrated Circuits*, 2nd ed. (Wiley, 2012).
9. Wolf, A. Wolf Lyapunov exponent estimation from a time series. MATLAB Central File Exchange https://www.mathworks.com/matlabcentral/fileexchange/48084-wolf-lyapunov-exponent-estimation-from-a-time-series (2014).
10. Sprott, J. C. *Chaos and Time-Series Analysis*. (Oxford University Press, 2003).
11. Ott, E. *Chaos in Dynamical Systems*. (Cambridge University Press, 2002).
12. Sukow, D. W., Gardner, J. R. & Gauthier, D. J. Statistics of power-dropout events in semiconductor lasers with time-delayed optical feedback. *Phys. Rev. A* **56**, R3370–R3373 (1997).
13. Ohtsubo, J. *Semiconductor Lasers: Stability, Instability and Chaos*, 4th ed. (Springer, 2017).
14. Schires, K., Gomez, S., Gallet, A., Duan, G. H. & Grillot, F. Passive chaos bandwidth enhancement under dual-optical feedback with hybrid III-V/Si DFB laser. *IEEE J. Sel. Top. Quantum Electron.* **23**, 1801309 (2017).
15. Bouchez, G., Malica, T., Wolfersberger, D. & Sciamanna, M. Manipulating the chaos bandwidth of a semiconductor laser subjected to phase-conjugate feedback. *Proc. SPIE* **11356**, 113560Y (2020).
